# How to create a scientific journal from scratch – the European Journal of Psychiatric Trainees

**DOI:** 10.1192/j.eurpsy.2023.208

**Published:** 2023-07-19

**Authors:** M. J. Santos

**Affiliations:** Mental Health Department, Hospital Prof. Doutor Fernando Fonseca, Amadora, Portugal

## Abstract

**Abstract:**

In this workshop we aim to discuss the ins and outs of publishing in psychiatry with practical guidance from both sides of the couch.

The executive editors of the European Journal of Psychiatry (Drs Asilay Seker, Mário J. Santos, Luís Fernandes) will talk about the experience of starting a journal from scratch with very limited resources and share practical tips with the participants about publishing for ECPs by ECPs.

**Disclosure of Interest:**

None Declared

